# Implementation assessment in confidential enquiry programmes: A scoping review

**DOI:** 10.1111/ppe.12604

**Published:** 2019-12-17

**Authors:** Hemali Jayakody, Marian Knight

**Affiliations:** ^1^ National Perinatal Epidemiology Unit University of Oxford Oxford UK

**Keywords:** confidential enquiry, health care, peer review, quality of health care, surveillance

## Abstract

**Background:**

Response should be a key part of maternal death surveillance and response (MDSR) programmes, which include confidential enquiries into maternal deaths. The programmes investigate avoidable factors in maternal deaths and make recommendations for improving maternity care. There is a gap in information on how these recommendations are transformed into practice.

**Objective:**

To explore the methods used to assess the implementation status of recommendations made in confidential enquiries into maternal deaths and other health outcomes.

**Data sources:**

We searched PubMed, Web of Science, CINAHL, and Google Scholar databases and general web for grey literature using the “Arksey and O’Malley framework” in all major scientific databases and search engines.

**Study selection and data extraction:**

An initial screening was followed by extraction of information using a data chart. Variables in the chart were based on the response component of maternal death and surveillance systems.

**Synthesis:**

Information collected was summarised using content analysis method.

**Results:**

We reviewed 13 confidential enquiry systems into maternal deaths. Many confidential enquiries into maternal deaths published reports with their recommendations and dissemination often involved national‐level scientific presentations. Only five reports provided strategies for implementing the recommendations. Follow‐up of previous recommendations was routinely published in only two reports. However, impact assessment of recommendations on other health outcomes was found only in the UK.

**Conclusion:**

There is a gap in monitoring the response generated by confidential enquiries into maternal deaths. Actions to develop this are therefore needed.

**Synopsis**

1 **Study question**

Explore the assessment of implementation status of recommendations made in confidential enquiries into maternal deaths and other health outcomes.

2 **What's already known**

Quantitative and qualitative review of maternal deaths through confidential enquiries lead to a set of recommendations. Implementing recommendations will result in reducing preventable maternal deaths.

3 **What this study adds**

Many confidential enquiries into maternal deaths use extensive procedures to make recommendations and disseminate them. However, the response component of maternal death and surveillance systems appears poorly developed. There is a gap in monitoring the implementation of recommendations generated by confidential enquiries into maternal deaths.

## 1 BACKGROUND

The Maternal Death Surveillance and Response (MDSR) programme is a massive scale quality improvement project in maternity care. On a regular basis, often annually, country‐level reports are produced and this information is shared with service providers and policy makers (stakeholders).^1^ Recommendations from confidential enquiry reports—one type of MDSR programme—are based on quantitative and qualitative assessment of maternal deaths. Multidisciplinary panels analyse each woman's care and combine lessons learned thematically to generate recommendations.^2^ Thereby, they translate data about maternal deaths into meaningful information for decision makers, the medical community, and the public.^3^

The response component in maternal death enquiries and surveillance systems plays a crucial role in ensuring sustainable changes to the health system. Implementing recommendations is a complex process.^4^ At a microlevel, participants of review meetings gain knowledge about avoidable factors which may change their practice by evoking a behavioural change. Recommendations themselves can be used to set goals, and their progress can be measured. Also, social pressure created from evidence of repeated guideline violations will encourage individuals to follow guidelines more closely.^2^ However, despite knowing the importance of following recommendations, many countries with established MDSRs still struggle to implement them.^5^ When recommendations are made on a national basis and therefore at large scale, they involve multiple levels of the health system, multiple interventions and engagement of multiple stakeholders at different time points are required to implement them. Implementation is thus a wide‐ranging and a shifting process^5^ and presents challenges.^6^

Knowledge of how to effectively incorporate a response component is therefore essential for those developing an MDSR programme, and there is a clear need for studies on the implementation component of maternal death reviews.^5^ We therefore aimed to explore methods used to drive implementation and systems available to assess the implementation status of recommendations made in confidential enquiries into maternal deaths as well as confidential enquiries into other health outcomes.

## 2 METHODS

A scoping review is a suitable method to explore broad areas with less distinct evidence and to summarise available interventions and programmes. Although confidential enquiry into maternal deaths (CEMD) is a widely used method of maternal death reviews, the evidence available about the implementation of recommendation is sparse.^5^ We therefore followed the “Arksey and O’Malley framework” for conducting scoping reviews.^7^ Only English language documents were included.

### 2.1 Search strategy for articles

We searched for keywords and phrases “confidential enquiry”* AND “maternal deaths”/“maternal mortality” AND implementation status/recommendations/ follow up/ compliance/monitoring* in PubMed, Web of Science, CINAHL, and Google Scholar. The search was limited to articles published from 1 January 2008 to 15 November 2018. Citations resulting from the search were saved, and duplicates were removed.

### 2.2 Search strategy for the grey literature

The same keyword combinations were used in a general search engine (Google). The first 100 search hits from each keyword combination were retrieved and duplicate entries removed.^8^ In addition, all references and web page links in selected articles and web pages were visited to identify candidate documents for further assessment.

### 2.3 Documents selection for the review

**Figure 1:**
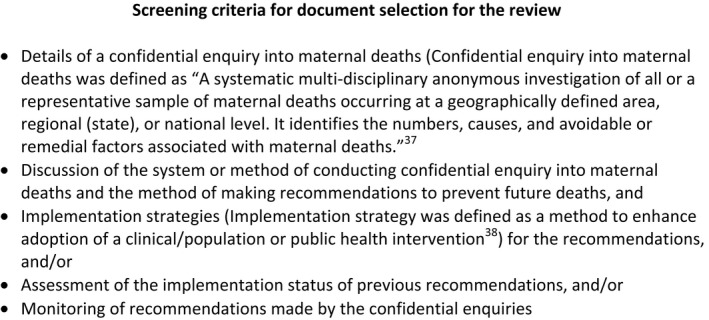
Screening criteria for document selection for the review

We used the criteria in Figure 1 to screen the articles for review. In instances where multiple versions of the same documents were available, we included only the latest version for data extraction (eg the most recent annual report).

A similar search was conducted for articles and documents on confidential enquiries into other health outcomes (excluding maternal deaths), and we applied the same screening criteria replacing the phrase confidential enquiry into maternal deaths with “confidential enquiry into health outcomes.”

### 2.4 Charting the data

Full articles were reviewed using a data chart.^9^ Variables in the chart were based on the response component of maternal death and surveillance systems.^3^ They included: who made the recommendations, delivery of recommendations, identification of the target group, follow‐up measures for recommendations, and evidence of impact or implementation assessment.

### 2.5 Collating, summarising, and reporting the results

We used Microsoft Excel to handle citations and databases creating a descriptive numerical summary for documents. We then conducted a content analysis of extracted data from articles and reports.^10^ Information collected from different sources on a particular confidential enquiry system (implemented at the national or subnational level) was triangulated and is referred to as a “system” hereafter.

## 3 RESULTS

**Figure 2:**
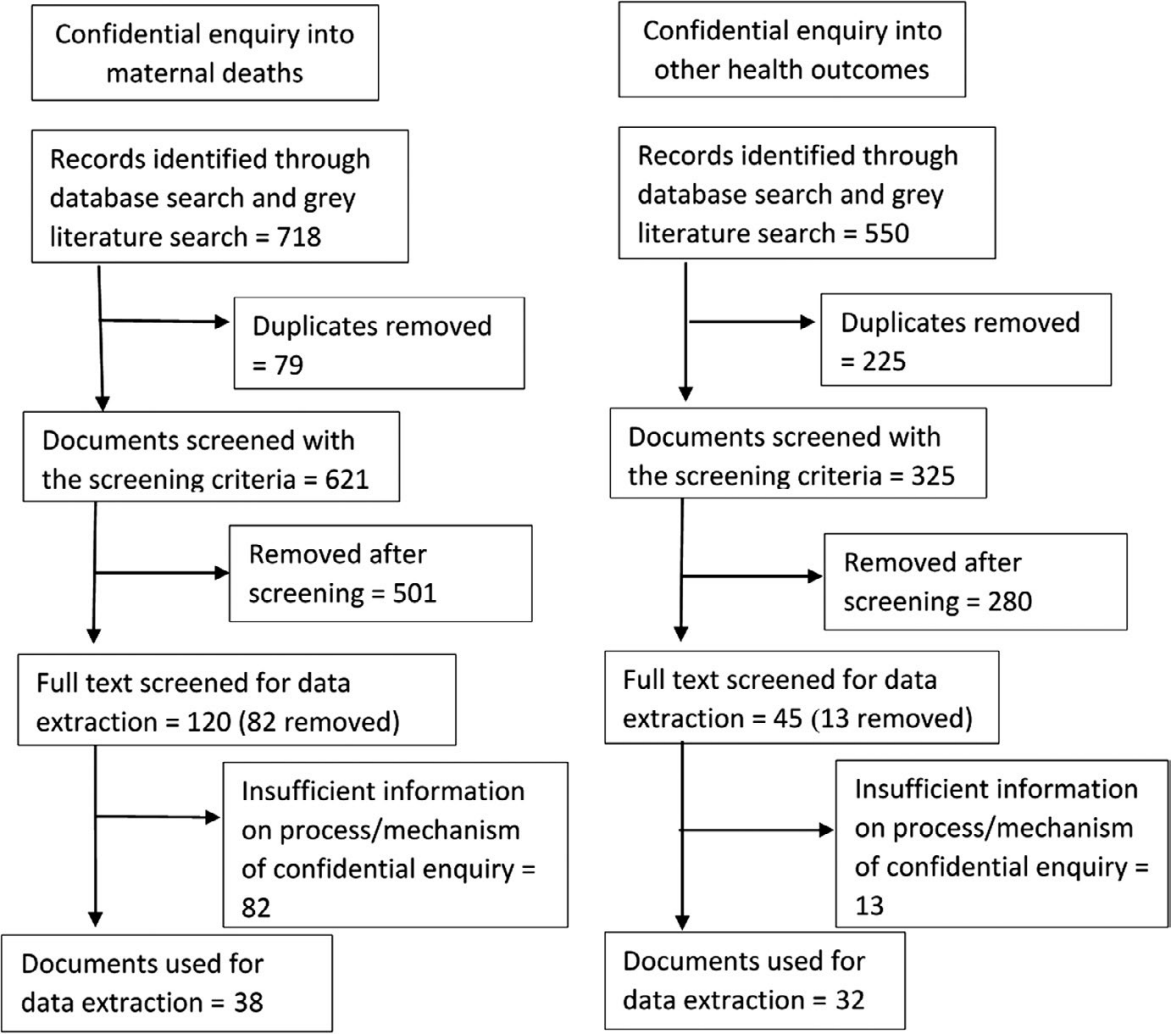
Search flow of documents into confidential inquiries into maternal deaths and other health outcomes (2008‐2018)

The initial keyword search yielded 621 unique documents and articles. Following the initial screening, 120 documents and articles were identified for further review. Information was extracted from 38 unique documents. There were 14 reports, 17 original papers, and five other journal articles (Figure 2).

The 38 documents reviewed described details of 12 national maternal death enquiries and one subnational level enquiry (Table 1).

**Table 1:** Summary of reviewed confidential enquires into maternal deaths

Country	Date of initiation of CEMD	Who makes recommendations	Method of dissemination	Frequency of dissemination	References
France	1996	Assessors of national confidential enquiry into maternal deaths which had wide representation	Report	Triennial	39
India ‐ Kerala	2004	Central review committee of Kerala foundation of obstetrics and gynaecologists—obstetricians and non‐obstetric clinical assessors	Report	“Periodically”	24
Japan	1995	Maternal death exploratory committee which consists of 15 obstetricians, 4 anaesthesiologists, 2 pathologists, emergency physician, and other specialists	As a journal article	Annual	21, 40
Kenya	2017	Assessors of maternal and perinatal deaths surveillance and review committee	Report	No information	22
Malawi	2009	National Committee on Confidential Enquiry into Maternal Deaths	Report	Annual	41
Malaysia	1991	CEMD national committee, which is led by a senior obstetrician	Report	Triennial	13
Moldova	2006	Confidential enquiry into maternal death committee which consists of clinical managers, department heads, and leading health professionals. The national committee consists of key individuals with authority	Report and a plenary session in a scientific congress for obstetricians	No information	14
Morocco	2009	National expert committee comprises of experts in obstetrics and gynaecology, anaesthesiology, public health, and one midwife	Report	Annual	11, 42
Netherlands	1998 (started in 1981)	Maternal mortality committee—consisting of 8 obstetricians and one maternal medicine internist. Dutch Society of Obstetrics oversees these activities	At the National Congress of the Netherlands Society of Obstetrics and Gynaecology. Case reports are published in the Netherlands Journal of Obstetrics and Gynaecology	Triennial	18, 43
New Zealand	From 2006	PMMRC—maternal mortality review committee consists of obstetricians, midwife, physicians, general practitioners, clinical nurse specialists, and perinatal psychiatrist	Annual report for both perinatal and maternal deaths	Annual	23
Nordic countries (Denmark, Finland, Iceland, Norway, and Sweden)	2010	Experienced group of clinicians forming a local audit group in each country	Presented at national meetings	No information	44
South Africa	1998	National Committee for Confidential Enquiry into Maternal Deaths consisting of experienced ministerial personnel representing obstetrics, midwifery, anaesthesia, and provincial representatives	Report on key recommendations and a combined policy brief on recommendations from maternity, infant, and child deaths	Annual	17, 20
United Kingdom	1952	The multidisciplinary writing group after reviewing cases in detail by consultant obstetricians, anaesthetists, midwives, psychiatrists, and if required by other clinical specialists	Report and a national launch meeting	Triennial until 2009. Annual from 2014 onwards	16

### 3.1 Making recommendations

Recommendations and key messages from confidential enquiries into maternal deaths were identified by the same panel who reviewed the deaths and identified the avoidable factors.^11^, ^12^, ^13^ Teams usually consisted of obstetricians, other medical practitioners, midwives, and occasionally pathologists. Two national confidential enquiry systems highlighted the importance of engaging “key individuals” with decision‐making capabilities in making recommendations; this practice ensured effective dissemination and prompt remedial action.^13^, ^14^ In the UK review system, the key emerging themes were identified at a multidisciplinary writing group meeting which was held after reviewing individual cases in detail.^15^, ^16^ In the South African CEMD, key recommendations were identified at the national and provincial stakeholder meetings to discuss maternal death enquiry data.^17^

### 3.2 Method of disseminating the recommendations

Commonly confidential reviews relied on an annual report to discuss the statistics and key lessons learnt. However, in four of the settings, publication of the report was complemented by presentations at scientific and/or at national report launch events.^14^, ^16^, ^18^, ^19^ Occasionally, review findings with similar underlying factors were presented in the same report. For instance, the Perinatal and Maternal Mortality Review Committee in New Zealand published a common list of recommendations to prevent maternal and perinatal deaths.^12^ In addition to the traditional report, the national committee on CEMD in South Africa produced a policy brief containing key recommendations made following infant, child, and maternal death reviews.^20^

### 3.3 Systems available to implement recommendations

Different systems facilitate the implementation process using different strategies. Most recommendations were addressed to stakeholders at different levels in the health system.^16^, ^20^, ^21^, ^22^ Some reports provided references to already available documents and guidelines which could be used as implementation tools.^16^, ^23^, ^24^ In the South African report, some chapters (eg hypertensive disorder, acute collapse, and pulmonary embolism) contain a guide for implementing the recommendations identified during the death review process. The guide contains activities to be implemented, the rationale for the stated action, who or what will act as facilitators or resources for implementation, and the level of the action within the health care system (eg district, clinical teams, civil society).^20^ For an example in the chapter on deaths due to embolism, substandard care for women was identified as a potential contributor (“motivator”). To address that, conducting drills was identified as the “action.” The “facilitator” for the action was district specialist teams, and the “level of action” was in the “district and ward PHC teams.” Similarly, multiple actions were identified at different levels, and they were presented with motivators (rationale), level of action, and facilitators. A list of actions pertaining to each recommendation was provided in the subnational report from India as a facilitator for implementation.^25^ A similar approach was seen in the confidential enquiries’ report from Kenya, where all recommendations were accompanied by corresponding actions and timeline indicators to monitor progress and targets to be achieved.^26^

In the most recent report from the UK, a list of auditable indicators based on recommendations was developed and presented in the report to monitor the progress of implementation of recommendations as an implementation tool.^16^ In Malawi, the Ministry of Health developed a maternal death review form to facilitate the implementation of the recommendations.^27^ At each hospital in Malawi, a focal point was identified to follow up the report recommendations and to share information on the progress at regular stakeholder group meetings.^28^

### 3.4 Assessing the impact of recommendations

Two brief desk reviews were published on the impact of recommendations from the South African and Malaysian review systems in 2014.^13^, ^17^ The South African review highlighted key achievements in maternity health following the publication of key recommendations. They include publication of guidelines and development of training modules to address “common causes of maternal deaths.” None of the reviews related the improvements to the recommendations using indicators or any objective measures. And neither of the papers described the exact methodology how of the review. Three reports provided key changes in maternity health provision since the publication of the previous report.^12^, ^16^, ^25^ For instance, the Perinatal and Maternal Mortality Review Committee of New Zealand provided a follow‐up of the progress since the first set of recommendations was published.^12^

### 3.5 Confidential enquiries into other health outcomes

Confidential enquiries into health outcomes are not limited to maternal deaths. We extended our review to assess documents published on other confidential enquiries into health outcomes. We located 32 documents and web pages which described a confidential enquiry process for health outcomes other than maternal deaths. Of these, 27 documents were from the United Kingdom and the remainder were from South Africa (two documents), Moldova, France, Mali, and Uganda. There were six reports and eleven articles which described confidential enquiries into health outcomes and five articles on assessing the follow‐up of recommendations made in confidential enquiries. Documents were generated from 19 different confidential enquiries conducted around the world.

The National Confidential Enquiry into Patient Outcomes and Deaths (NCEPOD) in the United Kingdom was established in 1989. NCEPOD conducts periodic surveys on specific topics in medical and surgical specialities.^29^ They disseminate their findings from each survey in a comprehensive report, a summary, and update the common recommendations’ report. “The themes and recommendations common to all hospital specialities” are a review of all recommendations published by NCEPOD studies. The recommendations were reviewed and organised according to common themes. This evolving document provides an easy reference to individual clinicians, trusts, and health boards to identify necessary improvements. In addition, NCEPOD provides a self‐assessment checklist and audit tool to assess the progress locally.^30^ The self‐assessment checklist contains the list of recommendations, and it enables the implementers to check on the changes that they have to make in their setting. Audit tools following NCEPOD enquiries are available for implementers to audit their cases following the introduction of recommendations from the enquiry. In addition, NCEPOD provides a forum to present findings of local audits using the tools at their launch events, encouraging the use of audit tools.

### 3.6 Method of dissemination

The main method of dissemination of key messages and recommendations as with CEMDs was through reports and journal articles. However, NCEPOD had report launch meetings on the day of release of the report and with presentations about the findings and recommendations at hospitals by invitation and discuss the implementation process with practising clinicians.^29^

### 3.7 Follow‐up studies of recommendations

There were several specific studies which examined the implementation of recommendations made following NCEPOD and National Confidential Enquiry into Suicides and Homicides (NCISH) reviews. Table 2 provides a summary of the five recently published implementation assessments identified in this scoping review. Three studies were conducted as national surveys with primary data collection, and the other two were regional studies.^31^, ^32^, ^33^ There was no consistent time gap between the dissemination of recommendations and at the time of onset of the study because these studies were by independent researchers. Four studies reported improvements in care either by following stated recommendations or by increasing attention to care because of the NCEPOD report.^32^ However, none of these studies were directly organised by the NCEPOD review system but were ad hoc work of enthusiastic clinicians.

**Table 2:** Confidential enquiries into other health outcomes and their follow‐up studies in the United Kingdom (UK)

Confidential enquiry	Follow‐up study setting	Scope of the study	Method of data collection	Conclusion	Time gap
National Confidential Inquiry into Suicide and Homicides (2001)	NHS in England and Wales	Key service recommendations from the NCISH report (2001)	Survey of service provision which enquired about availability of aspects of service and implementation of certain policies	Of the 12 recommendations, 7.2 per service were implemented by 2006	5 y since the publication^31^
National Confidential Enquiry—Acute Kidney Injury (2009)	All adult intensive care units in UK	Whether NCEPOD report was influential to their practice	Online survey	Low level of nephrologists’ input in intensive care units in UK	A few months since the publication of the report^32^
National Confidential Enquiry into Aneurysmal Subarachnoid Haemorrhage	All neurosurgical units in UK and Ireland	Key recommendations from the report and two other guidelines	A telephone interview with a registrar working on the ward	In majority of centres, recommendations were currently in practice. There were significant improvements in care	6 mo after publication of report^33^
National Confidential Enquiry into Parenteral Nutrition (2010)	Hospitals of Northern Nutrition Network in UK	Care of patients on parenteral nutrition	A simplified version of the audit tool used by NCEPOD inquiry team	Improved practice	3 y after the publication^45^
National Confidential Enquiry into Gastrointestinal Endoscopy (2004)	District general hospitals in North East England	Key recommendations from the NCEPOD report	A questionnaire to all patients who underwent endoscopic retrograde cholangiopancreatography (ERCP) during 3‐month study period	Good adherence to key recommendations	5 y after publication of report^46^

## 4 COMMENT

### 4.1 Principal findings

This scoping review revealed gaps in the response component of confidential enquiries into maternal deaths. The process of making recommendations and measures taken to disseminate them to target groups was extensive. Implementation strategies presented alongside recommendations provided some direction for converting them to into actions, but most systems lacked a process to monitor the progress of implementation of the recommendations.

### 4.2 Strengths of the review

Review is unique in its position in retrieving information on the response component of confidential enquiries into maternal deaths and other health outcomes. The information is useful for anyone who intends to improve the maternal death surveillance and response systems or anyone who intends to develop such a system from scratch. The response component is the most important in terms of achieving changes to the health system.

### 4.3 Limitations of the review

This scoping review was limited to documents published in the English language. Some reports and government documents pertaining to confidential enquiries into health outcomes were published in local languages, and some were not available in the public domain. However, we tried to minimise the gap by triangulating information collected from different sources about particular CEMDs. The use of grey literature searches was essential to cover the scope as most of the published work, and documents on confidential enquiries were available outside of formal scientific databases. Nevertheless, we found only 13 systems of confidential enquiries into maternal deaths in the database review. The small number reviewed may limit the generalisability of the findings. A comprehensive survey of all CEMDs with primary data collection could overcome this issue.

### 4.4 Interpretation

The overall goal of maternal death surveillance and response (MDSR) programmes is to provide information which can effectively guide actions to reduce maternal mortality.^1^ Findings from the confidential enquiry processes recommend individual‐ or organisational‐level changes to the health system. To facilitate the change process, implementation strategies are required.^34^ Targeted recommendations to stakeholders, implementation guides, links to available guidelines, and tools were found in some reports. There are proven and effective methods to achieve implementation targets.^4^ Implementation of recommendations needs to be monitored and evaluated.^5^, ^20^

In confidential enquiry processes, the response component should be able to monitor recommendations and its implementation and finally interpret the impact to inform future directions for recommendations.^6^ Assessing the impact of recommendations from CEMD was seen in few occasions. Commonly used strategies were ad hoc review studies and collecting recent changes to maternity care provision using desk review methods. However, none of these assessments were comprehensive and did not assess the intermediate outcomes of implementation of recommendations. The method used in New Zealand covers and follows up all recommendations. However, it also lacked the impact assessment.^23^ In addition, the audit tool used in NCEPOD enquiries is a comprehensive method to see the change of care due to implementation of recommendations but it is time‐ and resource‐consuming.

Most of the systems did not provide monitoring methods to facilitate changes. However, the “Saving Mothers’ Lives” 2018 report in the United Kingdom presented an audit mechanism to self‐assess the compliance with recommendations to be implemented alongside the recommendations.^16^ However, more time is required to determine the reception and the use of self‐audit tool. NCEPOD routinely publishes an audit tool with recommendations from each survey, to be used in patient care to assess the impact of the recommended quality improvements following the implementation of recommendations.^29^ The main feature of both tools is their ability to be used at different levels of the health system. Confidential enquiries usually recommend complex interventions which require multilevel action and monitoring mechanisms.^6^

Feedback from review data can influence the implementation process.^5^ Reports and meetings were the main methods of disseminating recommendations. Dissemination is the factor which will connect the results and recommendations to the necessary actions. Some systems used more than one method of dissemination by publishing a report and presentation at a national conference or a launch meeting. Executive summaries, policy briefs, lay summaries, scientific articles in reputed journals, and infographics were used to facilitate dissemination. Use of more than one method of dissemination is known to strengthen the effect of dissemination.^35^ Although recommended in the most recent technical guidance reports, we could not find evidence of the widespread use of modern methods of dissemination such as smartphone applications, text messages, and social media other than publications being made available on websites.^3^ Periodicity of reviews also impacts on the effectiveness of implementing recommendations. Some CEMDs publish reports once in three years; thus, the recommendation reaches service providers many years after the initial event. However, many systems have overcome from this by conducting local reviews early and issuing recommendations to the local level at an earlier stage.^17^, ^36^ In the maternal death surveillance‐response systems, apart from lay summaries and policy briefs engagement and dissemination efforts targeting the general public were minimal.

Commonly maternal death enquiries ended with the review, and no further follow‐up of recommendation implementation was attempted. Follow‐up of recommendations is essential to ensure accountability of the process.^1^ In general, we found there was no in‐depth periodic evaluation of recommendations identified from confidential enquiries. A few reports provided a summary of key changes in maternity care policies or guidelines since the last set of recommendations was published. This was limited to reporting of different activities rather than a periodic evaluation of implementation actions. A few ad hoc studies of the impact of recommendations on the care of women and patients (eg NCEPOD reviews) shed some light. However, they did not entirely meet the need for an in‐depth periodic evaluation in terms of improvements seen in communities, health care systems, and patient care.^37^ In in‐depth periodical evaluation, a regular process is available within the confidential enquiry system to assess the implementation level of recommendations considering inputs, processes, and outcomes.

### 4.5 Conclusions

In contrast to the rest of the confidential enquiry process, the response component of maternal death and surveillance systems appears poorly developed. Thus, there is a gap between making recommendations and implementing them. This leads to a lack of accountability regarding the implementation of a recommendation, potentially leading to reduced effectiveness of the MDSR process.^1^

We propose the following to improve the implementation of recommendations. Dissemination methods targeting different stakeholder groups and annual or regular reports with recommendations will be useful in getting the necessary messages to target groups. An implementation guide with detailed action and responsibility is a useful tool. Developing a mechanism to evaluate the implementation of MDSR recommendations periodically is essential. It could be a self‐assessment checklist on recommendations at the institutional level or an audit tool. Audit mechanisms such as NCEPOD tool are a comprehensive way of assessing the change of care due to implementation of recommendations or using a core set of indicators to assess the implementation are useful strategies to experiment for different settings. Engaging key individuals with decision‐making capabilities in the review process is another strategy to ensure that recommendations are implemented and to be informed about the impact of recommendations in health care provision.

Advocacy measures to ensure adequate funding and resources dedicated for implementing recommendations will ensure a definite path to improve the response component of CEMDs.
